# Supplementary figures and images for: Life Cycle Plasticity in Typhula and Pistillaria in the Arctic and the Temperate Zone
Source: Microorganisms. 2023 Aug 7;11(8):2028. doi: 10.3390/microorganisms11082028 (PMC10458498; doi:10.3390/microorganisms11082028)

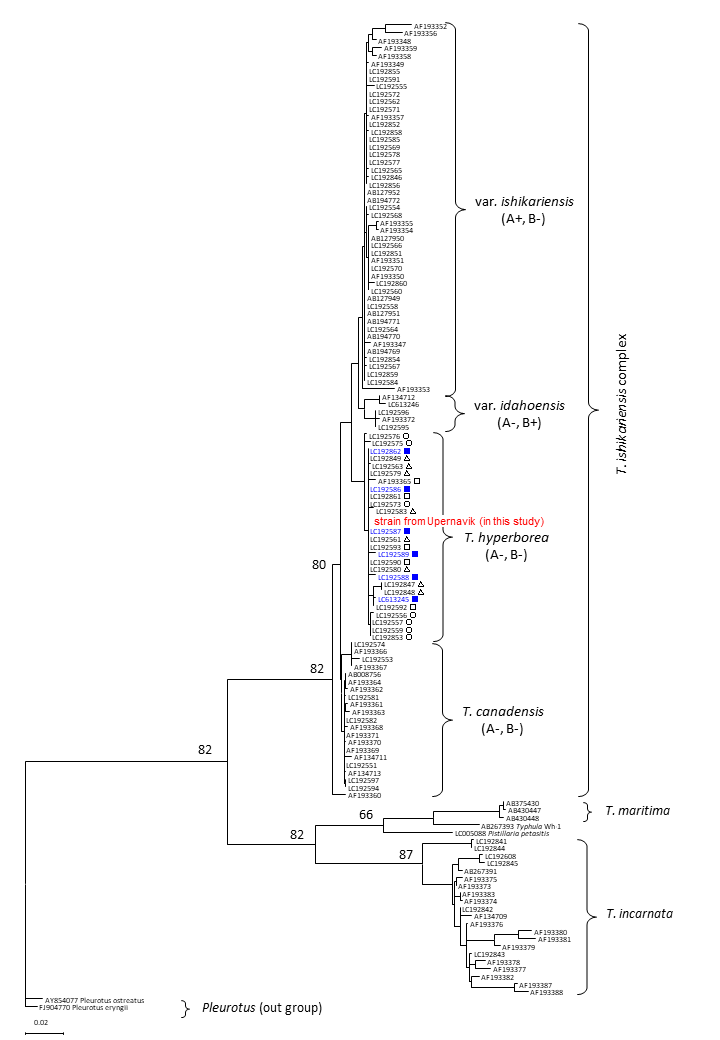

Supplement: Supplementary file 1 [file microorganisms-11-02028-s001.zip › microorganisms-2442792-supplementary.tif]
